# Efficient discovery of new medicine formulations using a semi-self-driven robotic formulator†

**DOI:** 10.1039/d5dd00171d

**Published:** 2025-07-17

**Authors:** Helena Ros, Youssef Abdalla, Michael T. Cook, David Shorthouse

**Affiliations:** a UCL School of Pharmacy 29-39 Brunswick Square London WC1N 1AX UK michael.t.cook@ucl.ac.uk d.shorthouse@ucl.ac.uk

## Abstract

We present the discovery of new medicine formulations using a semi-self-driven robotic formulator. Solubilising drugs is a significant challenge in the pharmaceutical industry, with the majority of active molecules in development for therapies being poorly soluble. The discovery of high solubility drug formulations, is a highly complex challenge involving the mixing of drugs with excipients in thousands of potential combinations. We have developed a self-driving laboratory process for the production, assessment, and optimisation of solubility of liquid formulations suitable for injectable medicines, and apply it to the example molecule curcumin. Our system discovered 7 lead formulations with high solubility (>10 mg mL^−1^) after sampling only 256 out of 7776 potential formulations (∼3%) in only a few days. Beyond presenting an efficient workflow for the optimisation and discovery of new liquid formulations, this work forms the basis for a more generalised optimisation workflow that could be applied to any formulation problem in the future, especially those where no prior information is known.

## Abstract 2

A self driving system is presented for rapidly optimising the solubility of drugs into medicines.

## Graphical abstract

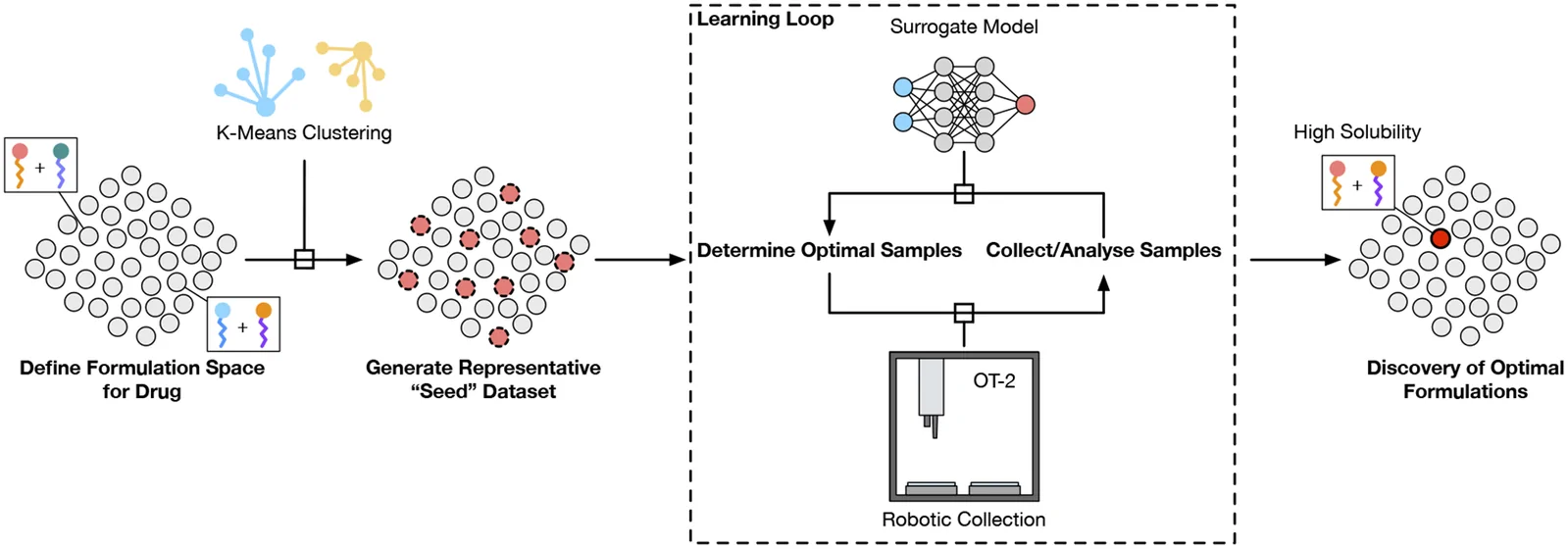

## Introduction

Integrating data science techniques with high-throughput laboratory automation allows for reconceptualization of scientific workstreams. Whilst automation has widely been used to increase the throughput of established assays or manufacturing processes, emerging techniques allow for automation processes to be focussed on discovery.^1–4^ Recent advances in this area include the mobile robot chemist, a robotic platform that performs experiments in a chemical laboratory, interprets the results of those experiments, predicts which experiment to perform next, then executes that experiment.^1^ Systems such as these which can proceed autonomously without human intervention are termed “self-driving” or “closed-loop” laboratories.^5,6^ These types of approach are delivering step-wise advancements in the chemical sciences using innovative batch and flow reactor systems. The most progressed examples of closed-loop discovery are in chemistry and materials science, including organic synthesis,^7,8^ catalysis,^1,9^ polymerisations,^10,11^ and battery applications.^3^ Whilst some processes exist as “fully self-driving”, defined as systems where human operators have almost no input to the workflow, a spectrum of self-driving laboratories exists, including hybrid “semi-self-driving” or “semi-closed-loop” systems where a bulk of the work is carried out in an automated fashion, with key components still requiring human intervention.^12^ This allows researchers to incorporate self-driving workflows without full commitment to the cost of robotics required in an entirely automated lab. Minimal examples of self-driving systems include materials discovery platforms using optical assessments that cost around $100.^13^ The opportunity of the autonomous laboratory is enormous and becoming increasingly accessible due to reducing costs of robotics and efficiency of machine learning (ML) techniques. Further development of self-driving laboratories across the sciences has enormous potential to expand current scientific frontiers.^5,14,15^

Pharmaceutical formulation is an area that could be revolutionised by self-driving laboratories. The development of a medicine from a lead drug compound requires multifactorial optimisation in a complex but well-constrained design space. Formulators require substantial experience and expertise to guide formulation using scientific principles in a field where the complexity of the final mixture of chemicals means that system behaviours are extremely challenging to predict from first principles. *In silico* tools have been developed to try and aid formulation decisions by prediction of crucial endpoints, such as solubility,^16,17^ however these predictions often do not hold in multicomponent mixtures and prediction across the entire chemical landscape is challenging. Furthermore, as complex modern medicines such as PROTACS^18^ appear in pharmaceutical pipelines, formulation scientists are not equipped with the *a priori* knowledge often used currently to solve problems of formulation. Whilst automation is often used as a tool for high-throughput analysis or manufacture in the pharmaceutical sector,^19,20^ the use of closed-loop approaches is limited. Lipids have been designed and synthesised for lipid nanoparticle-assisted RNA delivery using a closed-loop platform seeded from a literature dataset.^4^ Furthermore, the principle of closed-loop formulation has been demonstrated in consumer products.^21^ ML has been demonstrated to be a powerful tool for pharmaceutical formulation,^22^ but the necessity of high-quality datasets restricts the application of ML to well-studied problems. As such, experimental processes which pair automated workflows with data science techniques towards closed-loop automation are exceedingly valuable. For example, Bao *et al.* demonstrate the data-driven development of an oral formulation of lipid nanoparticles by pairing ML to rapid nanoprecipitation in a liquid-handling robot.^23^ However, the design space in this study was relatively small (∼1215 formulations) which allowed production of an initial dataset representing ∼10% of the possible formulations. Thus, there is a need to develop approaches which can solve formulations with a much larger number of possible permutations, which in principle could cover the plausible formulation space in its entirety.

One major challenge in formulation is the delivery of poorly-soluble actives, which represent 40% of the currently licensed medicines and 90% of small-molecule drugs in the development pipeline.^24^ Poor solubility in water can render a drug undeliverable, leading to product failure, or necessitate such large volumes of water to deliver that process, such as infusions over multiple hours, which require administration by healthcare professionals in a hospital setting. Regulatory constraints on the composition of medicines are such that innovation must occur with a limited selection of pharmaceutical excipients known to be safe, leading to formulators needing to innovate within a constrained chemical space.

This study reports the successful formulation of a poorly-soluble active molecule using an automated semi-self-driving workflow driven by an ML algorithm. The approach is driven without *a priori* knowledge or modelling to select excipients, instead using an unbiased experimental design to search a formulation landscape consisting of 7776 possible excipient combinations, of which we generate 256 across our entire study (3.3%). Automation and miniaturisation allows for rapid formulation, experimentally validating mathematical models as they are developed in a semi-self-driven manner. Lead medicines are identified by successful solubilisation of the target active (curcumin) which are then confirmed by a secondary manual batch preparation. Overall, a blueprint for pharmaceutical formulation using data-driven automation is presented.

## Results

### A semi-self-driving system for discovery of parenteral formulations

**Fig. 1:**
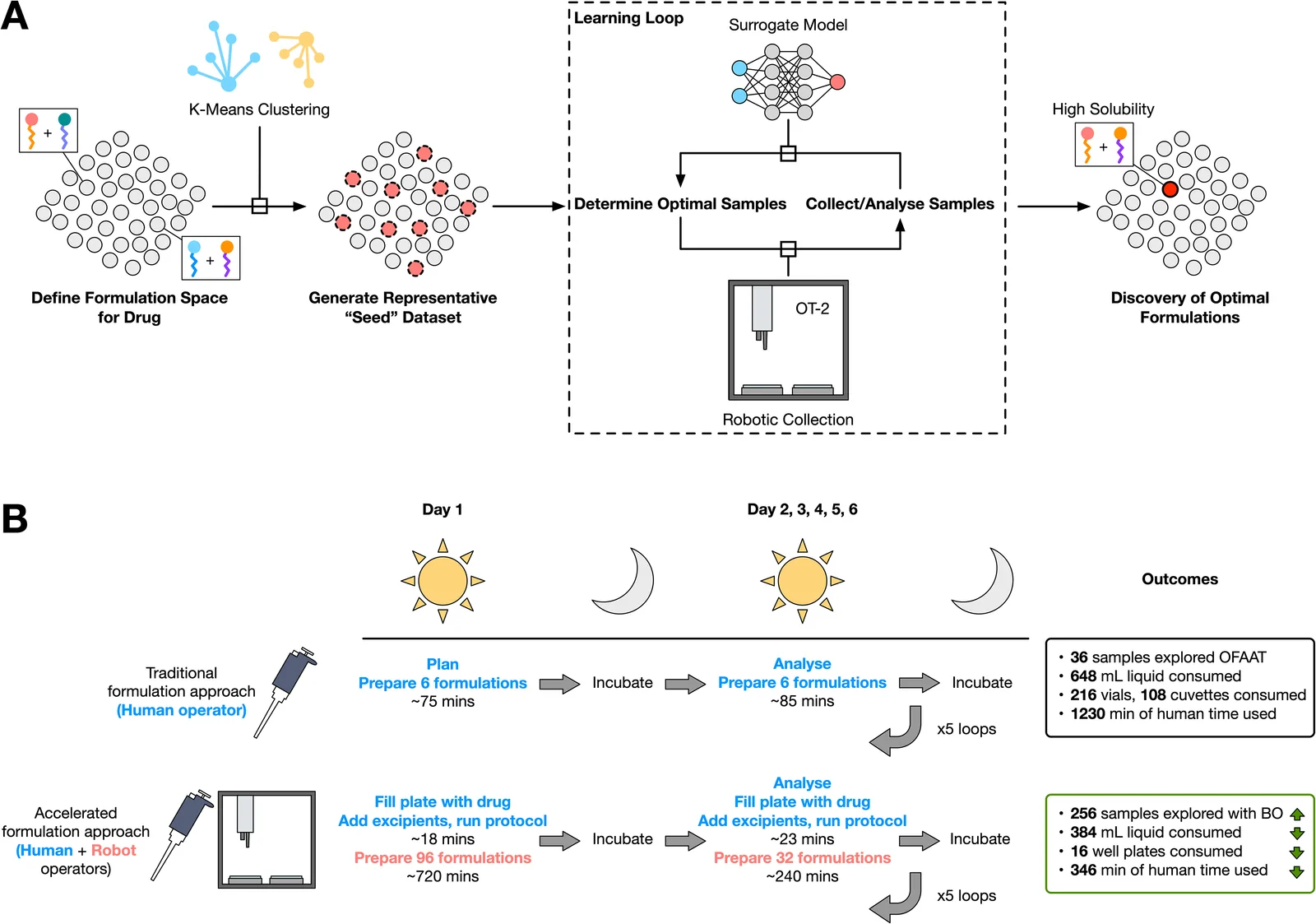
A workflow and timelines for semi-self-driven formulation of a molecule. (A) Schematic workflow for formulation discovery and optimisation. (B) Timeline for semi self-driving workflow compared to a traditional approach.

To generate and explore the parenteral formulation space for a given molecule, we employed a semi-automated, high-throughput sample preparation and analysis workflow (Fig. 1A). This workflow is capable of working with molecules for which there is no *a priori* knowledge, and so is highly adaptable to any molecule of therapeutic interest. The workflow involves defining the potential state space available for exploration (*i.e.* the potential combinations of excipients and concentrations thereof), generating and characterising a diverse “seed” dataset through applying *k*-means clustering to the state space, and then using this collected data to begin a series of Bayesian optimisation (BO) driven iterative and semi-automated learning loops to optimise the solubility of a molecule in an excipient mixture. This results in an efficient discovery of formulations optimised for solubility of the subject molecule. Sample preparation is carried out automatically through a liquid handling robot, the samples are centrifuged, diluted with a liquid-handling robot, characterisation is performed rapidly using a spectrophotometer plate reader, that absorbance data is handled automatically, a new round of experimentation is designed by an automated script running BO, which is then automatically coded to instruct the execution of that experiment by the liquid-handling robot. The only manual operation required is the loading of powder into plates, and transfer of well plates between devices. To demonstrate the applicability of this workflow, we applied it to an example of the poorly soluble molecule curcumin.^25,26^

Our designed workflow is significantly more efficient than manual equivalents (Fig. 1B). Within 6 days of operation the system can test 7 times as many formulations as a representative skilled formulator, whilst requiring only 25% of the human time. Furthermore, these samples are selected by BO and thus designed to optimise the formulation for solubility through a predictive model without need for human decision-making. Furthermore, in comparison to purely *in silico* approaches such as predictive models, the workflow delivers real experimental data to validate these predictions.

### Efficient discovery of seven novel curcumin formulations

Curcumin represents a good test case for our formulation workflow, as its colour allows easy by-eye confirmation of the veracity of generated formulations. We tested our system's ability to generate potential liquid formulations of curcumin by mixing it with 5 approved excipients/surfactants (Tween 20, Tween 80, Polysorbate 188, dimethylsulfoxide, and propylene glycol), which were available to the system in 6 percentages (0%, 1%, 2%, 3%, 4%, 5%) leading to a total of 7776 potential combinations. We applied our workflow, first generating a seed dataset of 96 diverse formulations (generated in triplicate) by *k*-means clustering and performing formulation using an OT-2 liquid handling robot with end-points determined spectrophotometrically. We then initiated 5 learning loops where 32 formulations each time were generated according to BO. Over the 5 loops, the algorithm was applied to optimise the solubility of curcumin, determined through absorbance in a plate reader. Assessing the final dataset, we applied a ML model to predict the concentrations of every possible formulation and used this to estimate the concentration range of the entire dataset (ESI Fig. S1†). Through this estimate, we determined that a dissolved concentration of 10 mg mL^−1^ was within the predicted top 0.1% of formulations and used this threshold to determine “highly soluble” formulations.

**Fig. 2:**
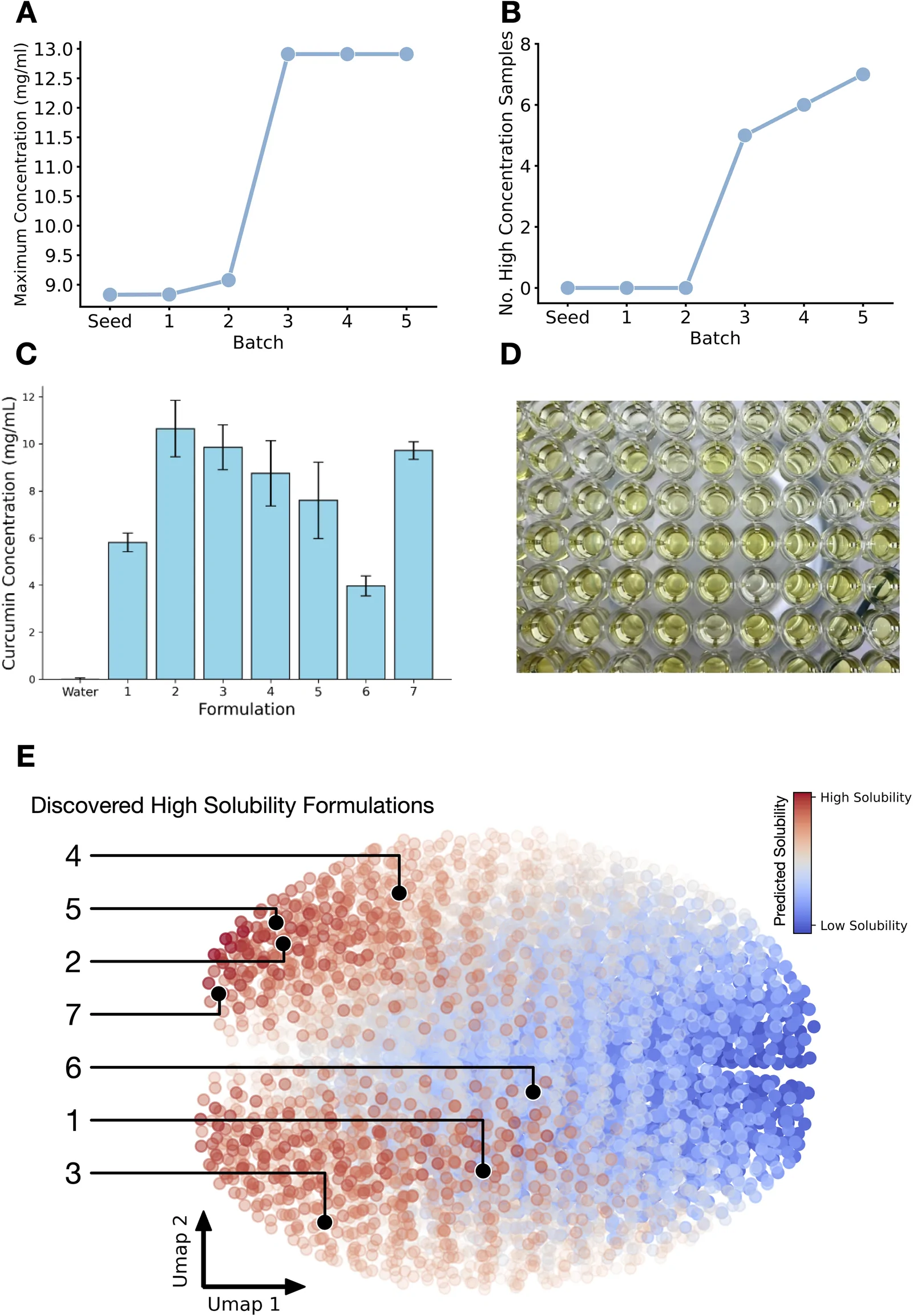
Novel formulations for curcumin discovered using our workflow. (A) Maximum solubility discovered at each step. (B) No. of samples with >10 mg mL^−1^ curcumin dissolved at each step. (C) Validated curcumin concentration of the 7 highest concentration samples discovered. (D) Image of well plate from final loop. (E) Solubility predictions of the final ML coloured on a umap projection of every possible formulation. Highlighted are the locations in state space where the discovered formulations sit.

The system quickly discovers highly soluble formulations (Fig. 2A) and increasing numbers of highly soluble formulations per loop (Fig. 2B). To validate these lead formulations, the discovered mixtures were manually generated in triplicate, and characterised again using absorbance spectroscopy. This validation confirmed three lead formulations (2, 3 and 7) maintained solubility levels around 10 mg mL^−1^ (Fig. 2C), predicted to be within the top 0.1% of all possible formulations (ESI Fig. S1†). Visual inspection of the well plate generated in the final loop confirmed a range of different solubilities, with a number of wells being bright yellow, indicating a high amount of curcumin has been dissolved (Fig. 2D). Finally, uniform manifold projection (UMAP) analysis demonstrates the formulation landscape discovered by our workflow (Fig. 2E). In this analysis, every possible formulation is represented and coloured by their predicted solubility according to the surrogate model used in the BO. Our discovered highly soluble formulations generally sit in the regions of the landscape predicted to have higher solubility, and formulations towards the mixed high and low solubility area of the landscape (formulations 1 and 6) were characterised as comparatively lower solubility compared to those within the high solubility region (formulations 2, 3, and 4). We interpreted this landscape by calculating a Pearson correlation of every excipient concentration against each UMAP dimension (ESI Fig. S2†), finding the concentration of all excipients except T20 is negatively correlated with UMAP dimension 1, and T20 is strongly negatively correlated with UMAP dimension 2. In particular, there is a tendency for formulations with a higher concentration of excipients to be more soluble (Pearson correlation of −0.82). We also compared the total amount of excipients in each formulation to the mean absorbance and found only a small but statistically significant correlation (*r* = 0.31, *p* = 4.4 × 10^−7^) (ESI Fig. S3†), suggesting that while excipient quantity influences solubility, the relationship is not straightforward.

**Fig. 3:**
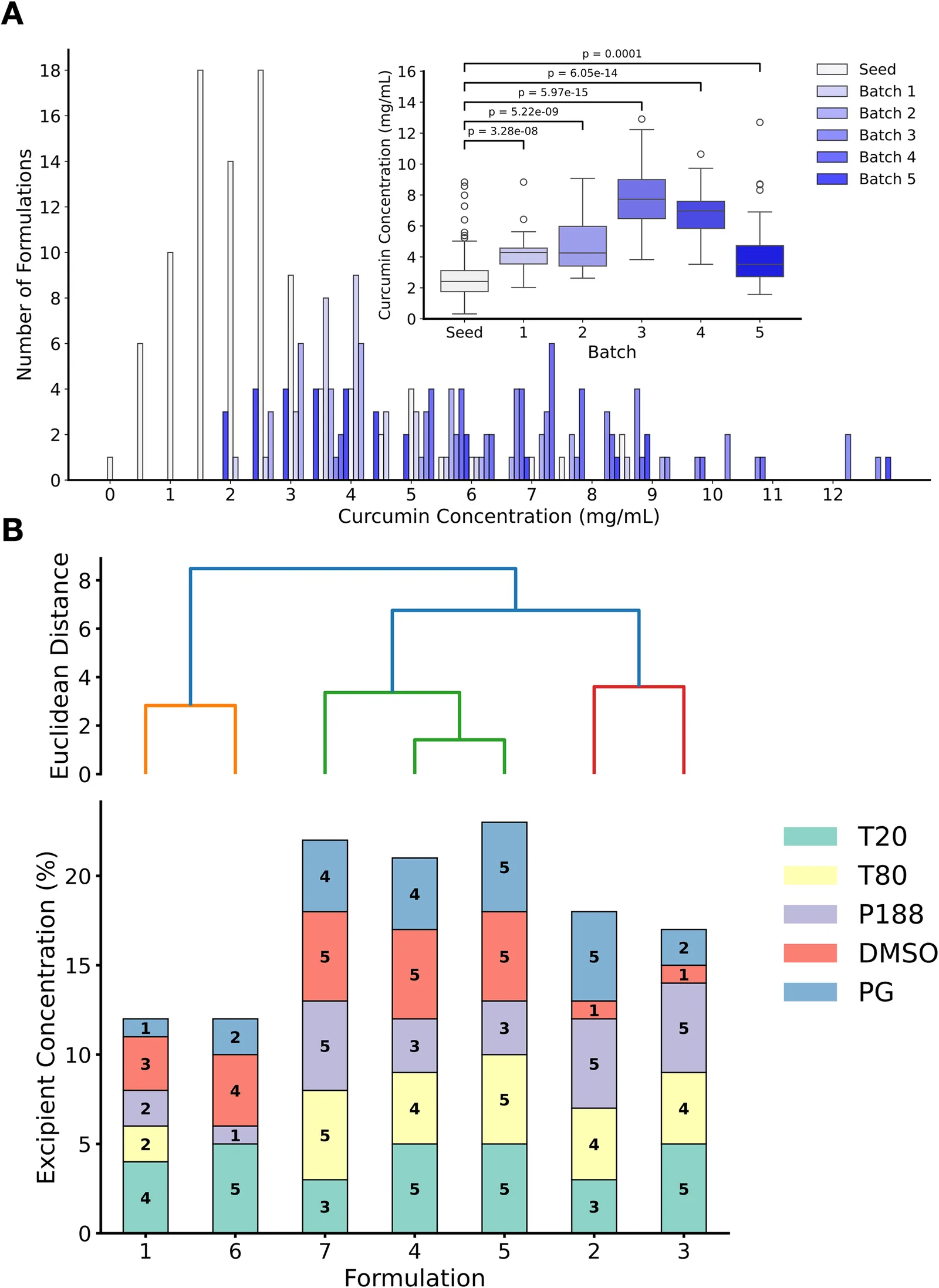
Assessment of Bayesian optimisation driven formulations discovered. (A) Per-round concentrations of curcumin, averaged to nearest 0.5 mg mL^−1^. Inset: curcumin concentration in each round. (B) Excipient makeup for each of the 7 high solubility formulations. Hierarchical clustering is shown above with nearest linkages colored separately. *P* values represent two-tailed Student's *t*-test.

During our workflow, we quickly discover formulations with a significantly higher solubility than those in our seed dataset from round 2 onwards (Fig. 3A). Comparing each loop to the seed dataset, which is optimised only for distance between samples, BO driven sample sets all contain significantly higher (Student's *t*-test *p* value < 0.05) average solubility formulations, demonstrating that our BO loops optimise the average of the explored samples to increase solubility as expected with our sample size of 32. We next explored the makeup of our 7 highest concentration lead formulations, performing hierarchical clustering on the excipient contents, we find that they broadly cluster into three categories, split predominantly by their overall concentration and amount of DMSO included (Fig. 3B).

### Bayesian optimisation is significantly more efficient than random approaches for discovering high solubility formulations

**Fig. 4:**
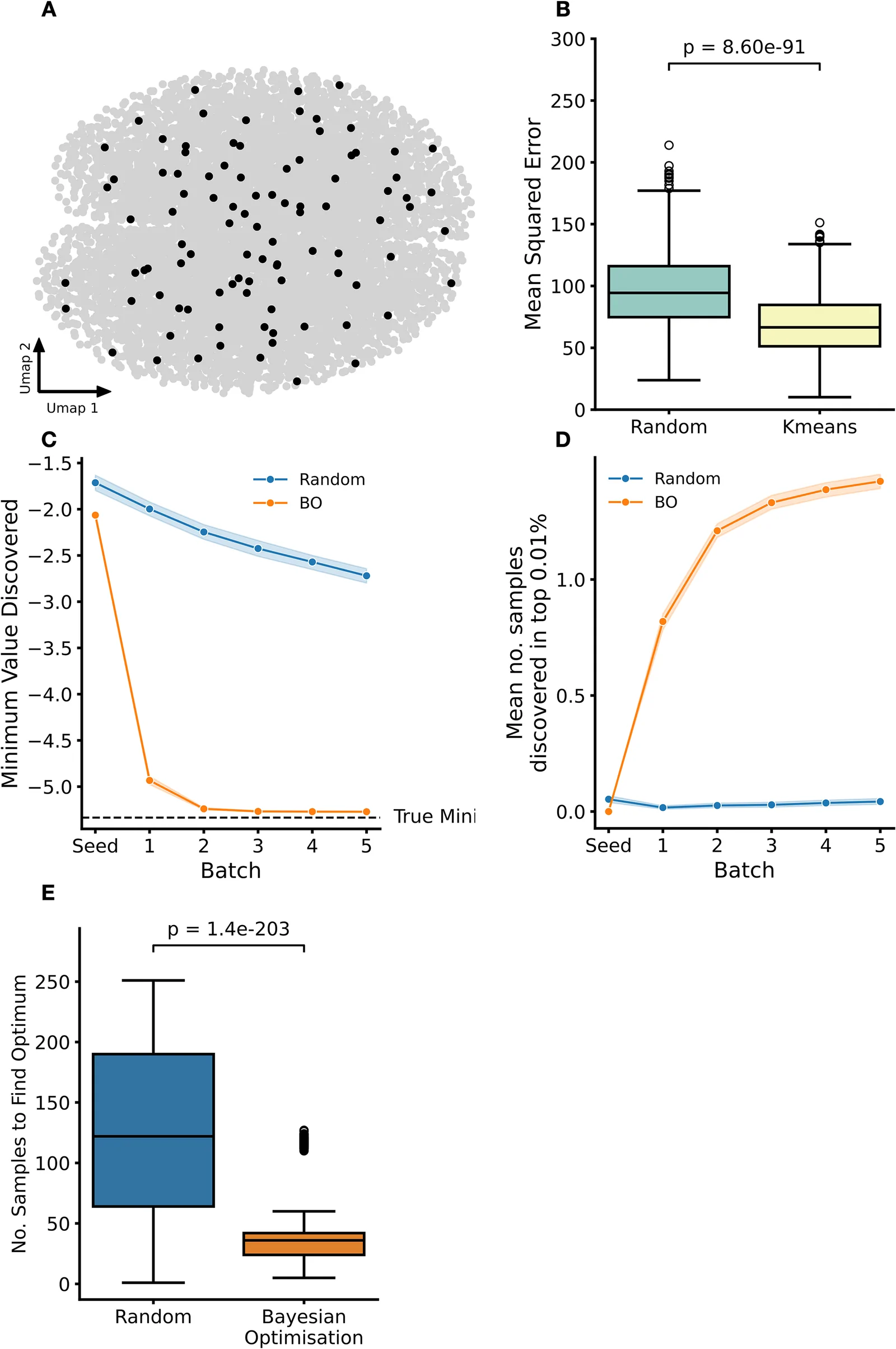
Assessment of our workflow compared to random sampling. (A) Umap of the formulation landscape with samples collected for seed dataset (determined using *k*-means clustering) highlighted in black. (B) Mean squared error for GPR fit to simulated data collected randomly *vs. k*-means clustering (*n* = 1000). (C) Minimum discovered value for 1000 simulated runs of BO compared to random sampling (D) No. samples discovered in the most optimal 0.01% for 1000 simulated runs of BO compared to random sampling. (E) Number of samples required to find the optimum for our collected dataset of 256. Samples are selected from the pool randomly, or using a Bayesian optimisation protocol, repeated 1000 times. *P* values represent two-tailed Student's *t*-test.

We next sought to demonstrate the value and efficiency of our BO driven approach over random sampling. Our approach involves the generation of a seed dataset used to initially train the model and we chose to use *k*-means to ensure diversity of starting samples. Assessment of the locations of the 96 starting samples on our umap projection of the complete dataset indicates that they are fairly evenly distributed across the landscape (Fig. 4A).

To more quantitatively benchmark our approach, we simulated a dataset with the same degrees of freedom. This dataset consists of 5 excipients with 6 degrees of freedom each (Totaling 7776 combinations), and their output solubility is a complex function consisting of all excipients, with a random noise term to ensure a true minimum. We first compared our *k*-means driven seed dataset sampling method to randomly sampling the same number of datapoints (Fig. 4B). A Gaussian process regressor (GPR) fit to 1000 repeats of *k*-means clustered *vs.* randomly determined sets of 96 datapoints generates significantly lower (Student's *t*-test *p* = 8.60 × 10^−91^) error models, demonstrating that this sampling method is quantitatively better than random sampling for training a surrogate model to initiate BO. Simulating BO *vs.* random selection protocols on this same dataset demonstrates that over 1000 protocols BO reaches a minimum close to the true minima reliably within 2 batches, having collected only 2% (160/7776) of potential samples (Fig. 4C). Similarly, BO rapidly discovers samples in the optimum 0.01% (the top sample) on average within 2 batches (160/7776 samples) compared to random sampling which almost never discovered and samples in the optimal 0.01% (Fig. 4D). Finally – to demonstrate that our collected data is descriptive and suitable for BO, we performed a post-hoc BO protocol on the 256 samples collected during our solubility workflow (Fig. 4E). We provided this set of samples to a BO algorithm, and allowed it to select from them one at a time measuring how many samples were required for the algorithm to find the optimum. We performed this 1000 times for a BO protocol based on our workflow, and with a seed sample size of 5, and compared it to randomly selecting samples, and find that BO is significantly (*p* = 1.40 × 10^−203^) more efficient at finding the true minimum in our data. This demonstrates that our workflow for seed selection and BO is effective and appropriate for this system, and that our collected data is able to be used predictively by a BO protocol.

### Gaussian process regression predicts accurately from data collected during the workflow

**Fig. 5:**
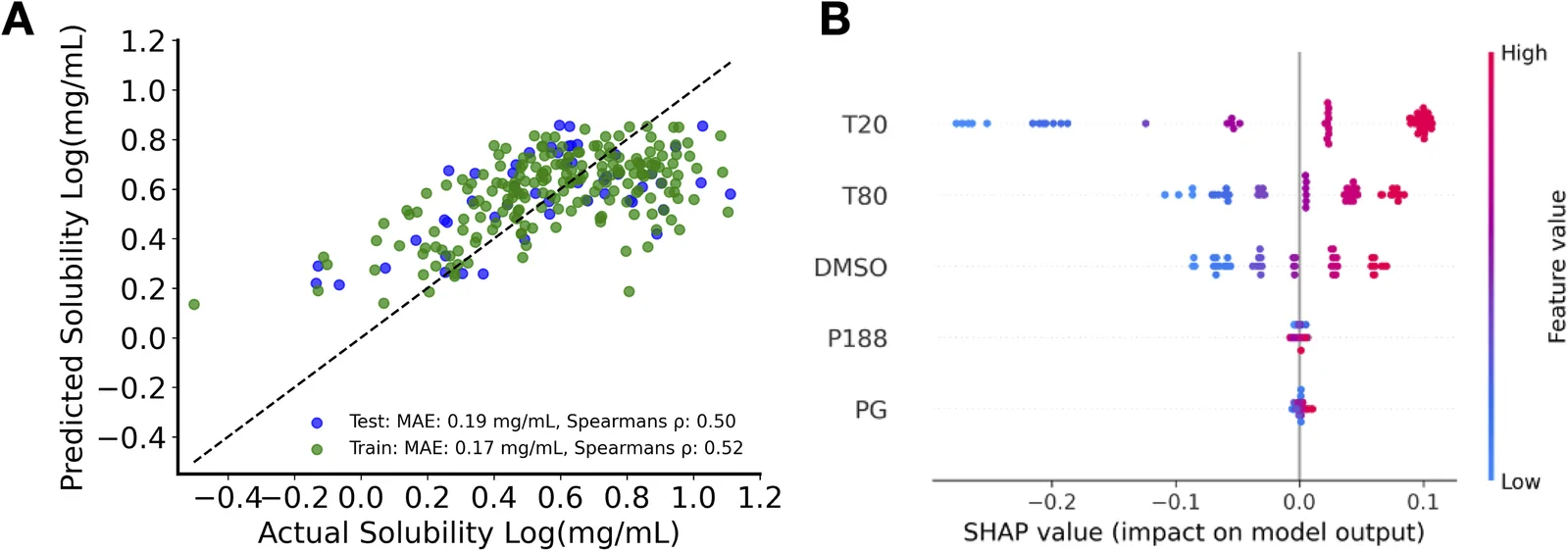
Assessment of GPR fit to collected solubility data. (A) Parity plot of log(mg ml^−1^) curcumin concentration showing real *vs.* predicted concentration of 256 formulations collected as part of our workflow. (B) Shapely (SHAP) analysis of the GPR fit to 256 formulations collected as part of our workflow.

Finally, to demonstrate that the data collected during our workflow is predictive, we demonstrate the accuracy of our surrogate mode, trained on only ∼3% of the total potential data (256/7776 samples). Parity plots demonstrate that our model is reasonably accurate, with a test set mean squared error of 0.19 mg mL^−1^. Spearmans rank analysis of the predictions shows a test set correlation coefficient of 0.51, indicating that the model can predict rank order of formulations somewhat. We note that the model appears to overpredict poorly soluble formulations, and slightly underpredict highly soluble formulations (Fig. 5A). Shapely (SHAP) analysis^27^ of the final model reveals that the proportion of Tween 20 is the most important predictor of solubility, with Tween 80 and dimethylsulfoxide also having a strong influence (Fig. 5B). Interestingly, Polysorbate 188 and propylene glycol (P188 and PG) did not appear to have a high importance, suggesting that they have a lesser effect on the solubility of the formulation. The observed accuracy gives some confidence that using a GPR on this data as a surrogate for BO is suitable.

## Discussion

We have demonstrated that a BO driven, robotically assisted, semi-self driving workflow is able to efficiently map a formulation space and discover lead formulations for a hard-to-dissolve molecule. With this workflow we have discovered 7 novel formulations of the hard-to-dissolve molecule curcumin that are similar in concentration to reported highly concentrated formulations of this molecule investigated for medicinal use.^26,28^ We demonstrate that our surrogate model is able to generate predictive insights from the collected data, and that our novel workflow discovers samples close to an optimum significantly more efficiently than random approaches. SHAP analysis demonstrates that the impact on solubility of our test molecule is mostly constrained to changes in only 3 of the 5 excipients, and so future expansions to this workflow aiming to increase solubility further would utilise this information to explore wider ranges of only these 3 molecules.

Our workflow requires significantly less time, fewer plates and materials, and can sample more formulations in the same time period compared to a traditional approach, and represents a near tenfold efficiency increase compared to traditional formulation workflows. Moreover, with the affordability of the setup used in this process (including the use of an entry-level liquid handling robot), this workflow is cost effective to establish.

A major limitation of our current system is the noise present in samples collected. High standard deviations between experimental repeats demonstrate inaccuracies likely induced by the liquid handling robot, such as droplet retention on pipette tips, incomplete mixing of viscous excipients, and inconsistences in manual plate handling, all of which can be overcome with development and improvement of protocols and adaptations to our workflow. This noise significantly reduces the efficiency of the optimiser algorithm^12^ which is evident in our post-hoc analysis of samples, where ∼50 samples were needed on average to discover the minima of our data. We expect that a reduction in system noise, or more advanced Bayesian optimisation techniques incorporating the experimental noise will significantly accelerate our optimisation. We also note slight deviation (<5 nm) in peak maxima following determination of absorption spectra, which is small but may lead to a propensity to slightly undermeasure true concentration values. Furthermore, in the present study we only measure a single output (concentration), and further expansions of this work must explore multiple end-points to determine the Pareto fronts that trade off important pharmaceutical processing parameters such as solubility and stability, as has been performed in other self-driving laboratories for different purposes.^29,30^

Our work links formulation science, data science, and automation in a step towards significantly more efficient formulation design and discovery. Future strides must be made towards “closing the loop” by incorporating more advanced automated processes, further reducing manual load, but our results demonstrate the initial feasibility and efficiency of this process. Our workflow is applicable to any formulation task requiring the handling and mixing of liquids – which includes pharmaceutical use cases such as biologic formulations and lipid nanoparticles, as well as other formulation tasks such as those involving paints and inks. In conclusion, we demonstrate a semi-self-driving workflow where a liquid handling robot, formulation scientist, and surrogate ML model work in concert to discover novel liquid formulations with optimal solubility.

## Materials and methods

### Materials

Propylene glycol (PG, >99.9%) was purchased from Fisher-Scientific (UK). Dimethyl sulfoxide (DMSO, >99.9%), Tween 80 (T80), Tween 20 (T20) and curcumin were purchased from Sigma-Aldrich (UK). Poloxamer 188 (P188) was obtained from Thermo-Fisher (UK). Deionised water (dH2O) was used in all experiments and all chemicals were used as received.

### Seed data selection

A full factorial combination of five excipients – Tween 20, Tween 80, poloxamer 188, DMSO and propylene glycol – at six concentration levels (integers between 0 and 5%) was generated, resulting in a total of 7776 possible formulations. To efficiently explore the entire formulation space, *k*-means clustering implemented using scikit-learn^31^ was used to select 96 formulations most evenly distributed across the formulation landscape. *k*-means clustering was applied with a number of clusters of 96, and the formulations closest to the centroid of each cluster chosen to be part of the seed dataset.

### Solubility analysis

The 96 selected seed formulations were prepared using the Opentrons OT-2 liquid handling robot (server version 7.2.1), with protocols designed using the Opentrons python protocol API (version 2.17).

Stock solutions of 20% (w/w) Tween 20, Tween 80, and poloxamer 188, and 50% (w/w) propylene glycol in dH2O were prepared. 96 well-plates were filled with curcumin by use of a pocked steel dispenser, loading each well with a volume of 48 mm^3^ of curcumin. Stock solutions were then combined in the wells according to the selected formulations and prepared by the OT-2 in 96-well plates containing curcumin. For human-operated batch analysis, formulations were prepared at 5 mL volume according to the ratios described in the BO. Excess curcumin was then added. The prepared samples were incubated overnight on a plate shaker (SciQuip Microplate Shaker) at room temperature. Following incubation, the samples were centrifuged at 2000 rpm for 20 minutes. The supernatant was then diluted 1000-fold in a 50% (w/w) DMSO/dH_2_O solution.

Spectrophotometry was performed using the ClariostarPlus spectrophotometer (BMG LabTech) with absorbance spectra recorded between 200 and 1000 nm at 5 nm intervals. Maximum absorbance was observed at 435 nm at which there was no absorbance from the constituent excipients (Fig. S2†).

### Bayesian optimisation

All computational analyses were performed using Jupyter Notebook (version 7.0.8) running python3. Absorbance data obtained at 435 nm were used as input to a surrogate model. GPyOpt^32^ was used to perform BO, training a Gaussian process regressor. Loops were performed by training the surrogate model on the collected data (the first loop used our 96 “seed” samples), and the next data points for collection determined through maximising expected improvement using Thompson sampling – first described in ref. 33. After each round of experiments all collected data was used to retrain the surrogate model, and a further set of data points determined for collection.

### Lead formulation characterisation

Following 5 rounds of BO, 7 formulations with a curcumin concentration greater than 10 mg mL^−1^ were selected as lead formulations. These formulations were prepared at a 1 mL volume by hand and excess curcumin added. The samples were left to shake overnight, filtered, and analysed by the absorption method described previously.

### Umap

Umap was performed using the umap-learn python implementation.^34^

### Hierarchical clustering

Hierarchical clustering was performed on selected final formulations using scipy.^35^

### Statistical analysis

All statistical analysis was performed using scipy.^35^ All tests performed were two tailed Student's *t*-tests.

### Gaussian process modelling

A Gaussian process model was fit to our data using the GaussianProcessRegressor in scikit-learn.^31^ Data was log-transformed, before being split 80 : 20 in a training and external validation set. The training set was then hyperparameter optimised using 5-fold cross validation, and the optimal parameters used to retrain the model on the full training set. This model was then tested against the external validation set to generate the final score. Model interpretation was performed using Shapely analysis.^27^

## Author contributions

Helena Ros: investigation, software, writing – original draft preparation. Youssef Abdalla: writing – review and editing. Michael T Cook: conceptualisation, methodology, writing – original draft preparation, writing – review and editing. David Shorthouse: conceptualisation, methodology, software, writing – original draft preparation, writing – review and editing.

## Conflicts of interest

All authors are founders of the company Implexis Limited.

## Supplementary Material

DD-004-D5DD00171D-s001

DD-004-D5DD00171D-s002

## Data Availability

Final solubility data generated in this study is available in ESI,† and all code used and generated in this publication is available at www.github.com/shorthouse-lab/Ros_SDformulation, published at https://doi.org/10.5281/zenodo.15861258.
