# Supplementary material for: Efficient discovery of new medicine formulations using a semi-self-driven robotic formulator
Source: Digit Discov. 2025 Jul 17;4(8):2263–72. doi: 10.1039/d5dd00171d (PMC13576497; doi:10.1039/d5dd00171d)
Supplement: Supplementary file 1 [file DD-004-D5DD00171D-s002.pdf]

Supplementary Information (SI) for Digital Discovery.
This journal is © The Royal Society of Chemistry 2025

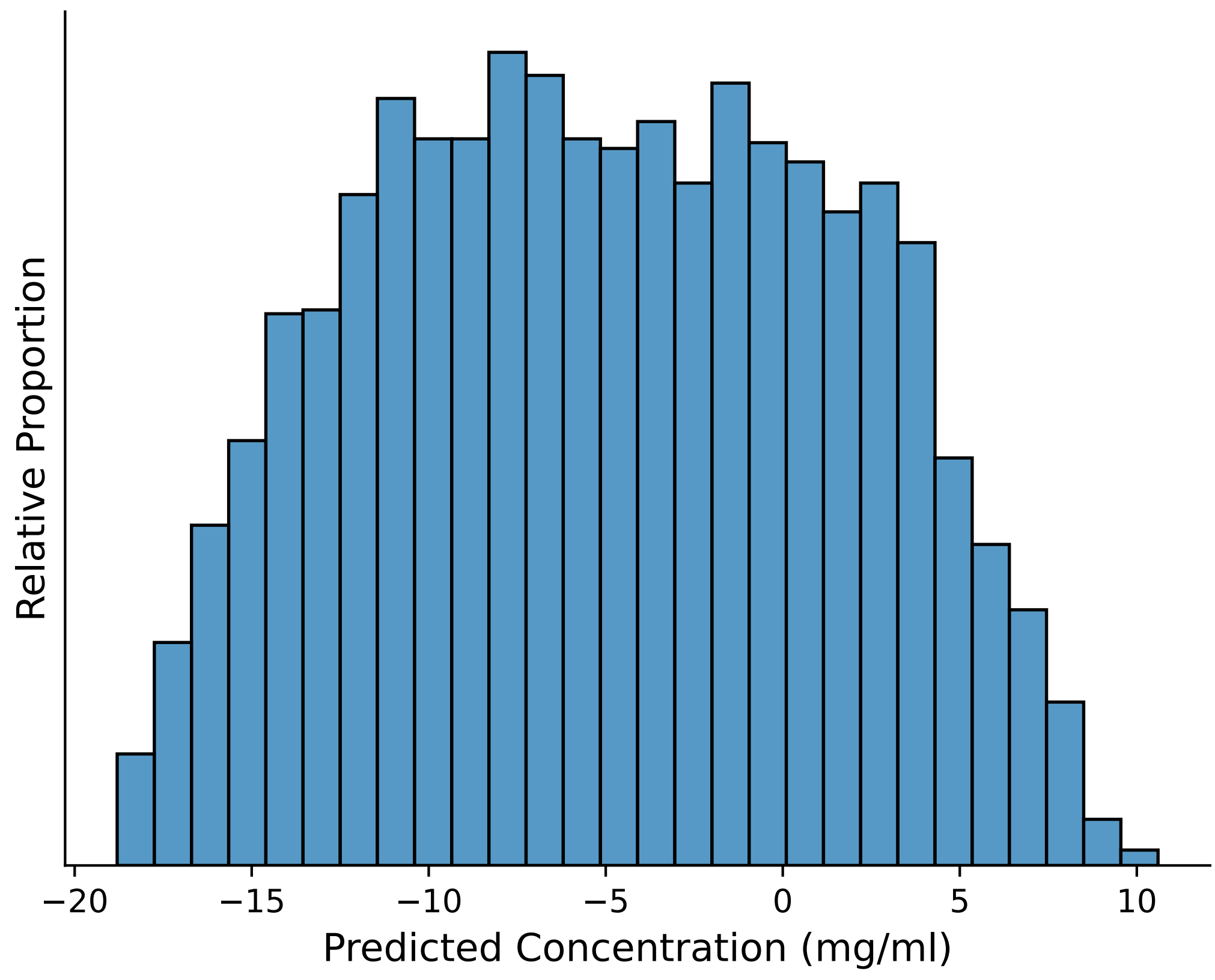

Figure S1: Predicted concentrations of all possible formulations in our landscape of 7776

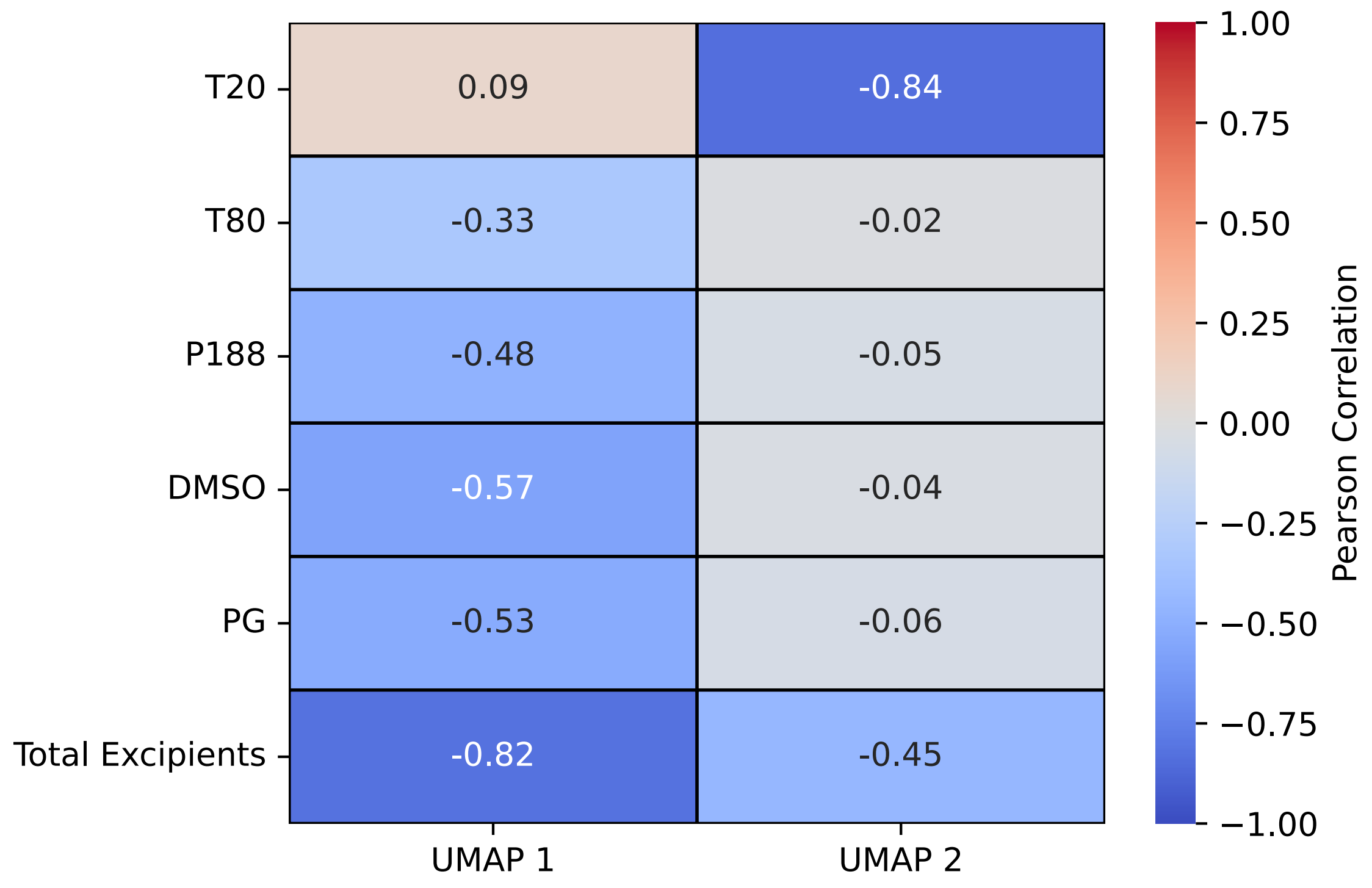

Figure S2: Pearson Correlation for each excipient amount against each UMAP dimension

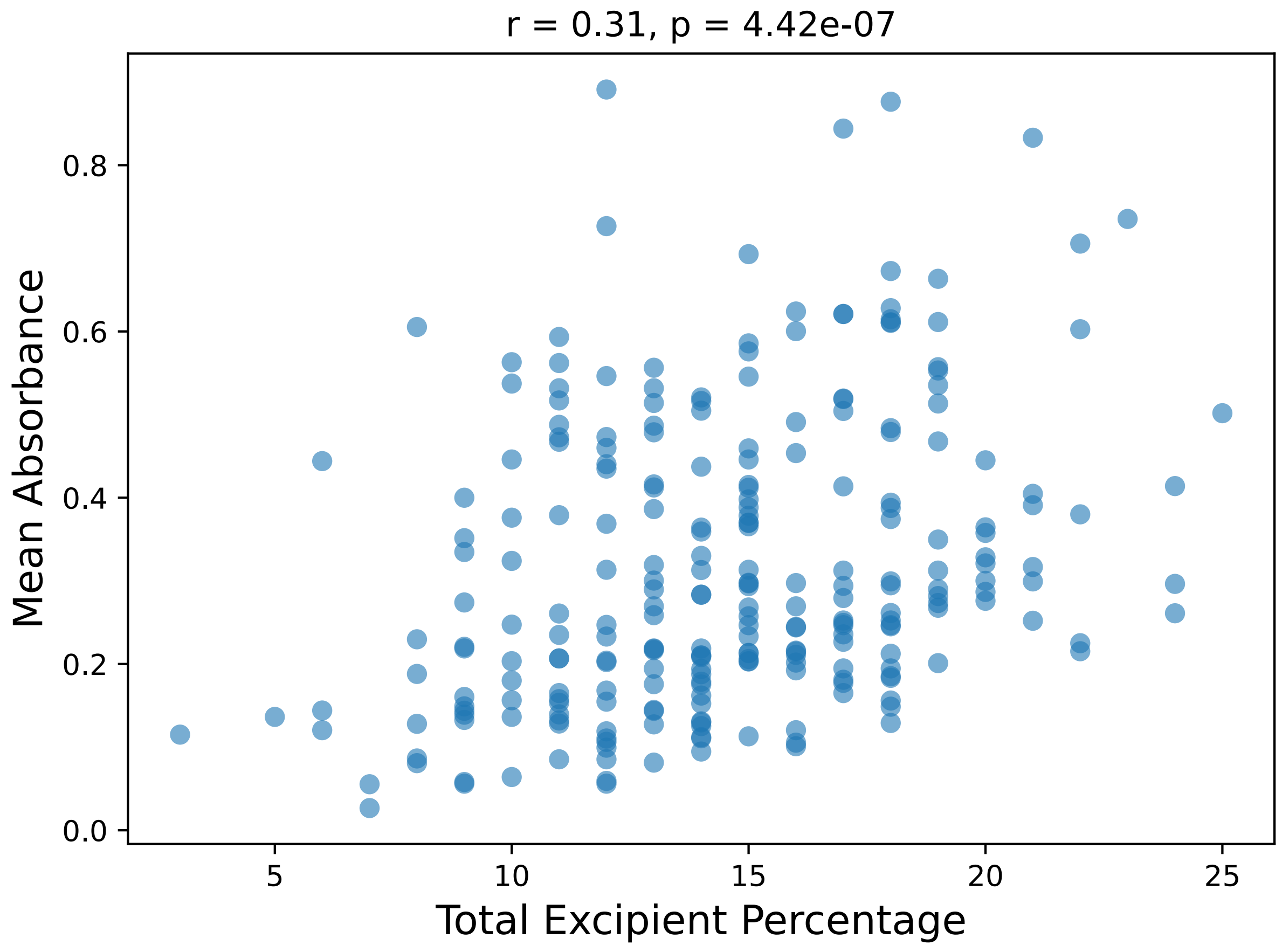

Figure S3: Pearson correlation between excipient percentage and mean absorbance per formulation

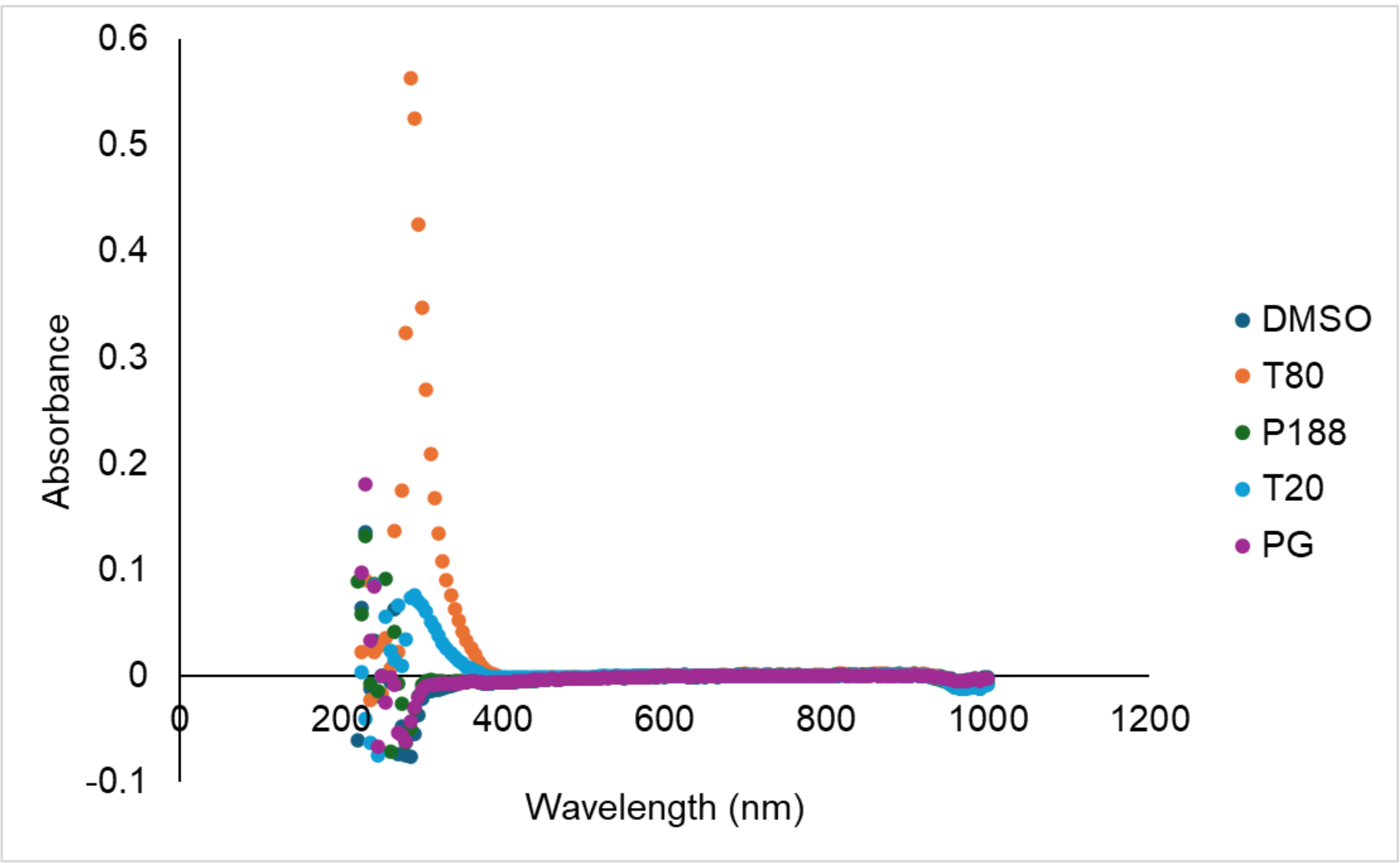

Figure S4: Absorption spectra for the excipients included in the generated formations
